# The influence of job satisfaction, resilience and work engagement on turnover intention among village doctors in China: a cross-sectional study

**DOI:** 10.1186/s12913-020-05154-0

**Published:** 2020-04-06

**Authors:** Xuewen Zhang, Liyan Bian, Xue Bai, Dezhong Kong, Li Liu, Qing Chen, Ningxiu Li

**Affiliations:** 1 Department of Health and Social Behaviour, West China School of Public Health and West China Fourth Hospital, Sichuan University, Chengdu, 610041 China; 2 School of Integrated Traditional Chinese and Western Medicine, Jining Medical University, Jining, 272067 China

**Keywords:** Rural health human resource, Village doctors, Resilience, Job satisfaction, Work engagement, Turnover intention

## Abstract

**Background:**

As the gatekeepers of rural residents’ health, teams of village doctors play a vital role in improving rural residents’ health. However, the high turnover of village doctors, both individually and collectively, threaten the stability of village medical teams. This research evaluated the influence of job satisfaction, resilience, and work engagement on the village doctors’ turnover intention, and explored the mediating role of work engagement and resilience between job satisfaction and the turnover intention of village doctors in China.

**Methods:**

A quantitative study using a self-administered questionnaire containing mostly structured items was conducted among village doctors with a sample size of 2693 from 1345 rural clinics in Shandong province, China, during May and June 2019. All variables including demographic characteristics, job satisfaction, resilience, work engagement and turnover intention were based on available literature, and measured on a 5- or 6-point Likert scale. Such statistical methods as one-way ANOVA, bivariate correlation, exploratory factor analysis (EFA), and Structural Equation Modelling (SEM) were used.

**Results:**

Up to 46.9% of the subjects had a higher turnover intention and more than 26.3% of them had a medium turnover intention. The job satisfaction of village doctors could not only have a direct negative effect on turnover intention (β = − 0.37, *p* < 0.001), but also have an indirect effect through work engagement (β = − 0.04,*=*< 0.001). Meanwhile, work engagement also had a direct negative impact on turnover intention (β = − 0.13, *p* < 0.001), and resilience had an indirect negative impact on turnover intention through work engagement (β = − 0.09, *p* < 0.001). The above results of this study strongly confirmed that job satisfaction, resilience, and work engagement were early, powerful predicators of village doctors’ turnover intention.

**Conclusion:**

According to the results, the following should be taken seriously to improve job satisfaction: reasonable and fair income, effective promotion mechanism, fair social old-age security, reasonable workload, and strong psychological coping mechanisms for work stress. The turnover intention of village doctors could be reduced through improving job satisfaction, resilience and work engagement.

## Background

In 2010, China began to implement integrated management of rural health services, and built standardized village clinics in villages, fundamentally changing the practice mode of village doctors, that is, village doctors must enter village clinics to practice diagnosis and treatment, and they are no longer allowed to operate clinics. Village doctors have been changed from self-employed to public service providers, playing a basic net role in China’s rural health care system. They are responsible for providing rural residents with life-cycle care involving prevention, treatment, health protection and rehabilitation, as well as two-way referral between village clinics and urban general hospitals, so that village doctors are the indispensable gatekeepers of health for the vast majority of rural residents in China [1]. With a rural population over 900 million, village doctors in China conduct a much broader scope of work compared to other countries. With the rapid development of China’s economy in recent years, the overall quality of village doctors, their service facilities, and the impartiality and accessibility of health services for rural residents have been continuously improved [2].

Yet village doctors are confronting serious challenges and pressures brought by some reformist policies concerning the medical and health system. The new national essential drugs system requires village doctors to use essential medicines and sell them to patients at zero-profit purchase price, shifting village doctors’ income from drug prices to local government compensation. The low level of government compensation and the delay in obtaining it have greatly reduced the income of village doctors. In 2014, village doctors’ average monthly income in Beijing, an economically developed area in China, was only 1293.07 yuan (166.637 euro) [3]. Meanwhile, In rural areas there are many serious dilemmas cannot be ignored, such as urgent demand for medical care, poor working conditions and environment, unsmooth promotion mechanism, limited training opportunities, lack of supervision and competition system, aging village medical team, and shortages of new doctors with professional knowledge and comprehensive quality [4], low income and rural residents’ distrust of their medical qualifications and medical technology make the occupational environment of village doctors increasingly severe [5]. All these factors have been confirmed to have an impact on village doctors’ burnout and subsequently turnover [3, 6]. The detection rate of job burnout among village doctors was as high as 68.6, 45.3% of which had high turnover intention [7]. Recently, China’s Henan province witnessed two serious cases of village doctors’ collective turnover, one involving 36 doctors and the other 28 doctors. As an important part of China’s medical and health service team, when village doctors leave collectively, rural medical institutions may face a “stall” and rural residents may be trapped in incurable difficulties, resulting in more serious social consequences [1, 8].

Turnover intention refers to an employee’s option to voluntarily vacate their work in a certain period of time [9]. Research on turnover can be traced to 1958 when March and Simon established the participant determination model [7, 10]. Subsequently, scholars have developed a series of theoretical models on turnover research; although the emphases of such models are different, they all state that turnover intention is the main cognitive precursor of turnover behaviour and has a strong explanatory power [11, 12]. Compared with turnover behaviour, turnover intention can better reflect the real management level of an organization. Based on this, this study found that it is more meaningful to explore turnover intention than actual turnover behaviour. Hence the turnover intention of village doctors, rather than their actual behaviour, is the theme of this study.

Since the training of doctors requires a long-term education and practice [13], high turnover tendency may lead to huge transition costs and serious loss of patients’ confidence, which is a serious problem for the stability of hospitals and the medical system [14, 15]. Numerous studies have analysed the influencing factors of doctors’ turnover intention, including a country’s medical system, occupational environment, doctor-patient relationship, level of employment and alternative job opportunities, and other external environmental factors [3, 16–18]. Internal individual factors include gender, age, marital status and work ability [4, 19, 20]. The most widely studied job-related factors including working hours, salary levels, social security, job stress and burnout, emotional commitment, job autonomy, fairness of remuneration [21, 22] were always incorporated into studies to explore the comprehensive impact on turnover intention. However, these researches have not involve the impact of resilience. At the same time, the existing studies have mostly used the t-test, Analysis of Variance (ANOVA) and chi-square test, as well as multivariate statistical analysis to analyse the influencing factors of turnover intention [3, 4, 16–22]. Structural Equation Modelling (SEM) can not only accurately and quantitatively measure the correlation between observed variables, but also track and deeply dig the correlation between latent variables, and even the causal relationship between measured variables. Therefore, this study adopted SEM to deeply explore the linear regression relationship between variables, which can make up for current studies’ limitations.

Job satisfaction, defined as a personal positive subjective evaluation or attitude towards all aspects of a work environment, is affected by many factors such as the work itself, work challenge, salary system, interpersonal relationships, working conditions, work motivation, organizational environment and so on, and has been typically considered to be the most representative antecedent variable that directly predicts turnover intention of health care providers [21, 23, 24]. Many empirical studies on medical staff have found that job satisfaction is negatively related to turnover intention, and influences it through direct and indirect paths [8, 24, 25]. Recently, a meta-analysis also demonstrated a significant negative correlation between job satisfaction and turnover intention among nurses [26]. In rural South Africa, improving job satisfaction by increasing wages, unblocking promotion channels, and improving guarantees was crucial to reduce the turnover intention of urban primary care physicians [27]. Furthermore, job satisfaction is often the mediator between other factors and turnover intention, for instance, through the mediating effect of job satisfaction, professional identity had an indirect negative effect on turnover intention among township health inspectors in China [7]. The doctor-patient relationship and work engagement also played an indirect role in nursing staffs’ turnover through job satisfaction in southern Italy [28].

Work engagement is defined as a positive, affective-motivational state of work-related well-being, with the characteristics of vigour, dedication and absorption [7, 29]. In countries or regions with limited medical resources, the work engagement of medical staff, in contrast to job burnout and high turnover intention, is recognized as an irreplaceable and much-desired organizational asset [30]. Personal characteristics such as psychological status, job identity and personality affect work engagement. Meanwhile, available work resources, organizational support and fairness, and other work characteristics are also highly positively correlated with work engagement [31, 32]. Accordingly, the outcome variables of work engagement include organizational variables such as organizational efficiency and performance, and personal variables such as job burnout and turnover intention [33, 34]. The total score of work engagement, vigour, dedication and absorption are negatively correlated with turnover intention. Some studies have explored the mediating role of work engagement in turnover intention [28, 35, 36]. Silvia De Simone et al. found that patient satisfaction had a negative effect on nurses’ turnover intention through the mediating effect of work engagement in southern Italy [35]. A cross-sectional and correlational study in a Portuguese hospital revealed the mediating effect of job engagement between social support, job satisfaction and turnover intention [37].

Yet even in the same working environment, facing the same pressure and adversity, not every village doctor will have job burnout or turnover intention. This can be explained by the concept of individual resilience, which refers to a person’s ability to recover from traumatic or painful events and achieve good adjustment and higher development [20]. Research have shown that individuals become stronger, more confident and more productive by experiencing stressful events and overcoming them through resilience [38]. In addition, resilience may not alleviate the stress experienced by village doctors, but it can improve their ability to overcome stress and predicament, thereby improving job satisfaction and work engagement, and reducing job burnout and turnover tendency [39]. A national survey of nurses’ turnover intentions in South Korea found that resilience and work engagement mediated the effect of work satisfaction and burnout on turnover intention [40].

**Fig. 1:**
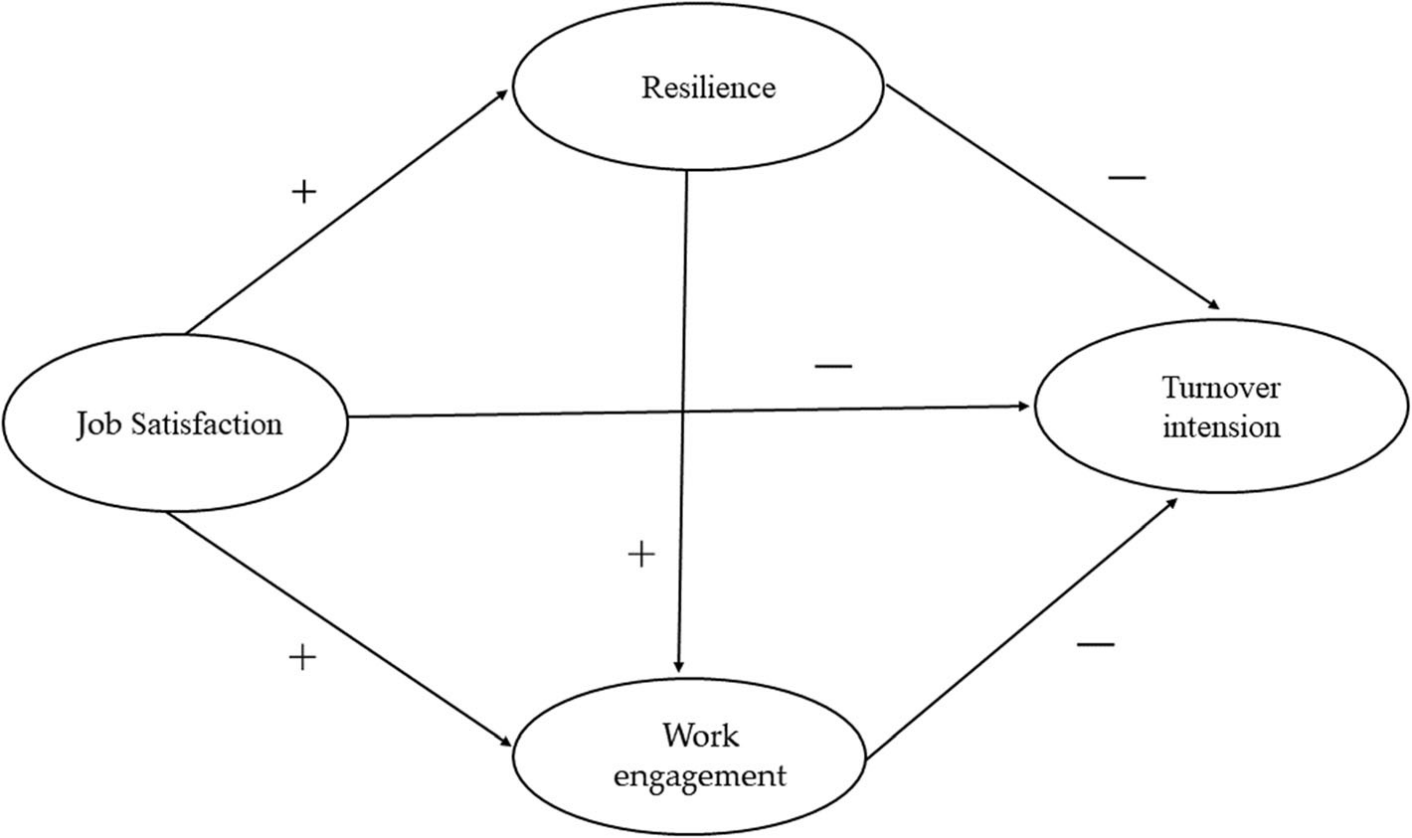
The theoretical model and hypotheses

Based on the above-mentioned theoretical analysis and empirical demonstration, we attempted to link the relationships among job satisfaction, resilience, work engagement and turnover intention, and a double mediator model was presented in Table 1 and Fig. 1. We assumed that job satisfaction, resilience and work engagement directly affect turnover intention. Meanwhile, through resilience and work engagement, job satisfaction has an indirect effect on turnover intention, and work engagement mediates the effect of resilience on turnover intention. So, this study aimed to verify the direct impact of job satisfaction, resilience and work engagement on turnover intention, and to analyse and quantify the mediating role of resilience and work engagement between job satisfaction and turnover intention among village doctors in China. This study is the first to consider and explore the influence of job satisfaction, resilience, and work engagement on village doctors’ turnover intention in China.

**Table 1:** The Theoretical Hypotheses

Hypotheses	
1. The village doctors’ job satisfaction has a direct negative effect on turnover intention	
2. The village doctors’ job satisfaction has a positive effect on resilience	
3. The village doctors’ job satisfaction has a positive effect on work engagement	
4. The village doctors’ resilience has a positive effect on work engagement	
5. The village doctors’ resilience has a negative effect on turnover intention	
6. The village doctors’ work engagement has a negative effect on turnover intention	
7. The village doctors’ job satisfaction has an indirect negative effect on turnover intention through the mediating effect of resilience	
8. The village doctors’ job satisfaction has an indirect negative effect on turnover intention through the mediating effect of work engagement	
9. The village doctors’ job satisfaction has an indirect positive effect on work engagement through the mediating effect of resilience	
10. The village doctors’ resilience has an indirect negative effect on turnover intention through the mediating effect of work engagement

## Methods

### Setting and participants

Jining in southwest Shandong province lags behind the province’s eastern part in terms of economic development. According to 2018 statistics, the total population of Jining is 8.375 million, of which the rural population is 3.5916 million. Jining has 6489 villages with 5307 village clinics. There are 11,715 village doctors, 870 fewer than in 2017, averaging 2.99 per village. The number of consultations in village clinics is 21,262,000, with an average annual consultation volume of 4006.41 per clinic [41]. As village doctors have a heavy workload, Jining city was taken as the research site to ascertain the turnover intention of village doctors and the influencing factors.

This cross-sectional study was performed among rural clinics in Jining from March to May in 2019. A hierarchical cluster random sampling technique was used to obtain the ultimate sample. Firstly, according to the level of economic development, Jining City’s 11 counties were divided into three layers: better areas, general areas, and poor areas. Secondly, a county was randomly selected from each economic layer as a sample county, i.e. three counties were selected altogether. Thirdly, all village doctors from the selected three counties were taken as survey samples.

The data was collected through self-administered questionnaires, including six parts besides the cover letters. In order to reduce survey bias, firstly, in the cover letters we stated the purpose and emphasized that the study was anonymous. Secondly, the questionnaires were distributed through cities, counties, townships and villages. At each level, there were people in charge of issuing, collecting and verifying questionnaires, thus reducing the loss and omission of questionnaires. The questionnaires were delivered to 1345 rural clinics through an official letter by Jining Municipal Health Commission. They were completed by 2789 village doctors and 2693 useable questionnaires were returned, i.e. effectiveness exceeded 96%.

### Measures

In the design of the questionnaire, this study referred to the National Residents Health Service Questionnaire designed and finalized by a panel of experts of the National Health Committee of the People’s Republic of China [42], and a series of items were revised and supplemented according to the working characteristics of village doctors. The main part of the questionnaire consisted of five parts: social demographic characteristics (age, gender, marital status, major, professional ranks and titles, salary, average weekly working hours, educational background, years of work), and questions related to job satisfaction, resilience, work engagement and turnover intention.

#### Job satisfaction

The Chinese version of the Medical Staff Job Descriptive Index and its related scale, with a Cronbach’s alpha coefficient of 0.828 [7, 43], was used to measure village doctors’ job satisfaction. It comprised eight items: workload, colleagues, superiors, environment and facility, promotion, income, social security, and training opportunities. A 6-point Likert scale ranging from 1 (highly disagree) to 6 (highly agree) was utilized to evaluate all these items, and all items were scored in the positive direction. The higher the single item or total score, the higher the village doctors’ satisfaction with their current work.

#### Work engagement

With a Cronbach’s alpha coefficient of 0.782, the Chinese version of the Utrecht Work Engagement Scale was used to measure the village doctors’ work engagement [7, 44]. It comprised 17 items, and 3 dimensions were represented by sub-scales: work dedication (5 items), work vigour (6 items), and work absorption (6 items). Using a 7-point Likert scale of 0 (never) to 6 (always), items were measured and scored, and the answers of each village doctor were merged into a summary scale. The higher the score, the higher the enthusiasm for medical work engagement [7].

#### Resilience

Resilience was assessed with the Connor-Davidson Elasticity Scale (CD-RISC), and the Cronbach’s alpha was 0.92 [45]. The CD-RISC with 25 items was a self-reported scale with good reliability and validity, and divided into five dimensions: ability, tolerance of negative emotion, acceptance of change, control, and spiritual influence. However, when using CD-RISC to measure the overall population of China, Yu Xiaonan et al. failed to confirm the original five-dimensional model by the statistical method of confirmatory factor analysis, and they instead found three dimensions of psychological resilience: tenacity (13 items), strength (8 items), and optimism (4 items) [46]. A 5-point Likert scale of 1 (complete disagreement) to 5 (complete agreement) was utilized to evaluate all these response to the items. The higher score, the stronger psychological resilience to cope with work pressure.

#### Turnover intention

Village doctors’ turnover intention was measured by the Chinese Turnover Intention Scale with 4 items, and the Cronbach’s Alpha coefficient was 0.659 [7]. The four items in the questionnaire were: “I often want to leave my present job”, “I often want to leave my present career”, “Recently, I often want to change my job”, and “I’ll probably find a new job next year”. A six-point Likert scale ranging from 1 (highly disagree) to 6 (highly agree) was utilized to evaluate all these items. The higher score indicated the more significant turnover intention.

### Statistical analysis

In this study exploratory factor analysis (EFA) was used to scientifically assess the responsibility and validity of the whole questionnaire. The socio-demographic characteristics of 2693 village doctors were examined by descriptive statistical method. Then, their job satisfaction, resilience, work engagement, and turnover intention were analyzed separately with descriptive analysis and the values of means and standard deviations (SD) were calculated. The correlation between the main observational variables was quantified with the Pearson correlation coefficient. Based on the above research results, the structural equation model (SEM) was used to further explore and quantify the relationship between the four dimensions: job satisfaction, resilience, work engagement, and turnover intention and a bootstrap-based maximum likelihood was applied in the SEM. Several indicators including adjusted goodness of fit index (AGFI), normed fit index (NFI), goodness of fit index (GFI), comparative fit index (CFI), Tucker-Lewis index (TLI) and incremental (IFI) were 0.90 or above, while root mean square error of approximation (RMSEA) was lower than 0.08 [7], which reflected an acceptable fit between the current data and hypothesized model.

### Reliability and validity

In accordance with the EFA results, the Kaiser-Meyer-Olkin (KMO) of this questionnaire was 0.826 greater than 0.70, indicating a better possibility of factor analysis. Bartlett’s test of sphericity was significant (*X*^2^ = 23,795.504, *P* < 0.001). For factor load analysis, the maximum coefficient of variation method was used for orthogonal rotation (varimax) to obtain the results of the factor load matrix after rotation. The eigenvalues of the four evaluation indexes were all more than 1, and the cumulative variance contribution rate was up to 80.353%. The load values of each item in the corresponding dimension were greater than 0.727, which indicated that the questionnaire structure validity was good. The Cronbach’s α of the whole questionnaire is as high as 0.848, indicating good reliability of internal consistency [7].

## Results

### Demographic characteristics of participants

The sociodemographic characteristics of the 2693 village doctors are shown in Table 2. Ranging from 19 to 76 years old, village doctors’ average age was 44.6 ± 7.3 years, and only 1.3% of them were under 30 years old. The vast majority only had technical secondary school education (68.2%), and 3.8% had middle and senior professional titles. 41.1% of respondents had worked between 20 and 29 years, 46.4% earned less than 2000 yuan per month, and 69.8% needed to work 60 h or more per week.

**Table 2:** Demographic characteristics of participants (*n* = 2693)

Socio–Demographic	N	%
**Gender**		
Male	1736	64.5
Female	922	34.2
Missing	35	1.3
**Age, Group**		
< 30 years	36	1.3
30–39 years	624	23.2
40–49 years	1302	48.3
≥ 50 years	685	25.4
Missing	46	1.7
**Professional ranks**		
Senior title	15	0.6
Mid-level title	86	3.2
Primary title	1306	48.5
No title	1166	43.3
Missing	120	4.4
**Years of work**		
< 10	87	3.3
10–19	830	30.8
20–29	1104	41.1
≥ 30	587	21.8
Missing	76	2.9
**Marital status**		
Unmarried	70	2.6
Married	2551	94.7
Missing	72	2.7
**Education background**		
University or above	71	2.6
Junior College	658	24.4
Technical secondary school	1836	68.2
High school education or below	91	3.4
Missing	37	1.4
**Monthly income (yuan)**^**a**^		
< 1000	311	11.5
1000–1999	939	34.9
2000–2999	769	28.6
≥ 3000	506	18.8
Missing	168	6.2
**Weekly working hours**		
< 40	390	14.5
40–59	336	12.5
≥ 60	1880	69.8
Missing	87	3.2

^**a**^ As of the date of this paper writing, the exchange yuan-euro exchange rate according to the People’s Bank of China was 0.1278

### Descriptive analysis of study variable

The total item scores of job satisfaction, resilience, work engagement and turnover intention were 32.48 ± 8.93, 74.01 ± 17.06, 66.14 ± 20.26, and 12.16 ± 6.09 respectively. The item scores contained in each dimension are shown separately in Table 3. According to the scores, 722 (26.8%) of village doctors had low turnover intention, 708 (26.3%) had moderate turnover intention, and 1263 (46.9%) had high turnover intention. Job satisfaction with workload (3.79 ± 1.50), promotion (3.74 ± 1.53), income (3.54 ± 1.55), and social security (3.70 ± 1.28) was lower than the other items.

**Table 3:** Item scores in job satisfaction, resilience, work engagement and turnover intention

Items	Mean ± SD
**Job satisfaction**	32.48 ± 8.93
Workload	3.79 ± 1.50
Colleagues	4.71 ± 1.30
Superiors	4.72 ± 1.37
Environment and facility	4.12 ± 1.44
Promotion	3.74 ± 1.53
Income	3.54 ± 1.55
Social Security	3.70 ± 1.28
Training opportunities	4.16 ± 1.28
**Resilience**	74.01 ± 17.06
Tenacity	37.53 ± 9.79
Strength	23.93 ± 5.66
Optimism	12.54 ± 3.06
**Work engagement**	66.14 ± 20.26
Work vigour	23.56 ± 7.02
Work dedication	19.7 ± 6.28
Work absorption	22.87 ± 7.63
**Turnover intention**	12.16 ± 6.09
Thought of leaving the organization you serve now	3.11 ± 1.59
Thought of leaving this industry	3.11 ± 1.62
Looking for a new job recently	3.05 ± 1.64
Looking for a new job next year	2.88 ± 1.61

### Correlations of study variables

The correlation coefficients between four main observation variables all reached a level of significance. Job satisfaction, resilience, and work engagement negatively correlated with turnover intension, and the three positively correlated, as shown in Table 4.

**Table 4:** Correlation coefficients among study variables

Items	Job Satisfaction	Resilience	Work Engagement	Turnover Intention
**Job Satisfaction**				
**Resilience**	0.45^a^			
**Work Engagement**	0.41^a^	0.67^a^		
**Turnover Intention**	−0.39^a^	−0.24^a^	−0.27^a^	

^a^Test for trend: *p* < 0.01

### Test of study model

**Fig. 2:**
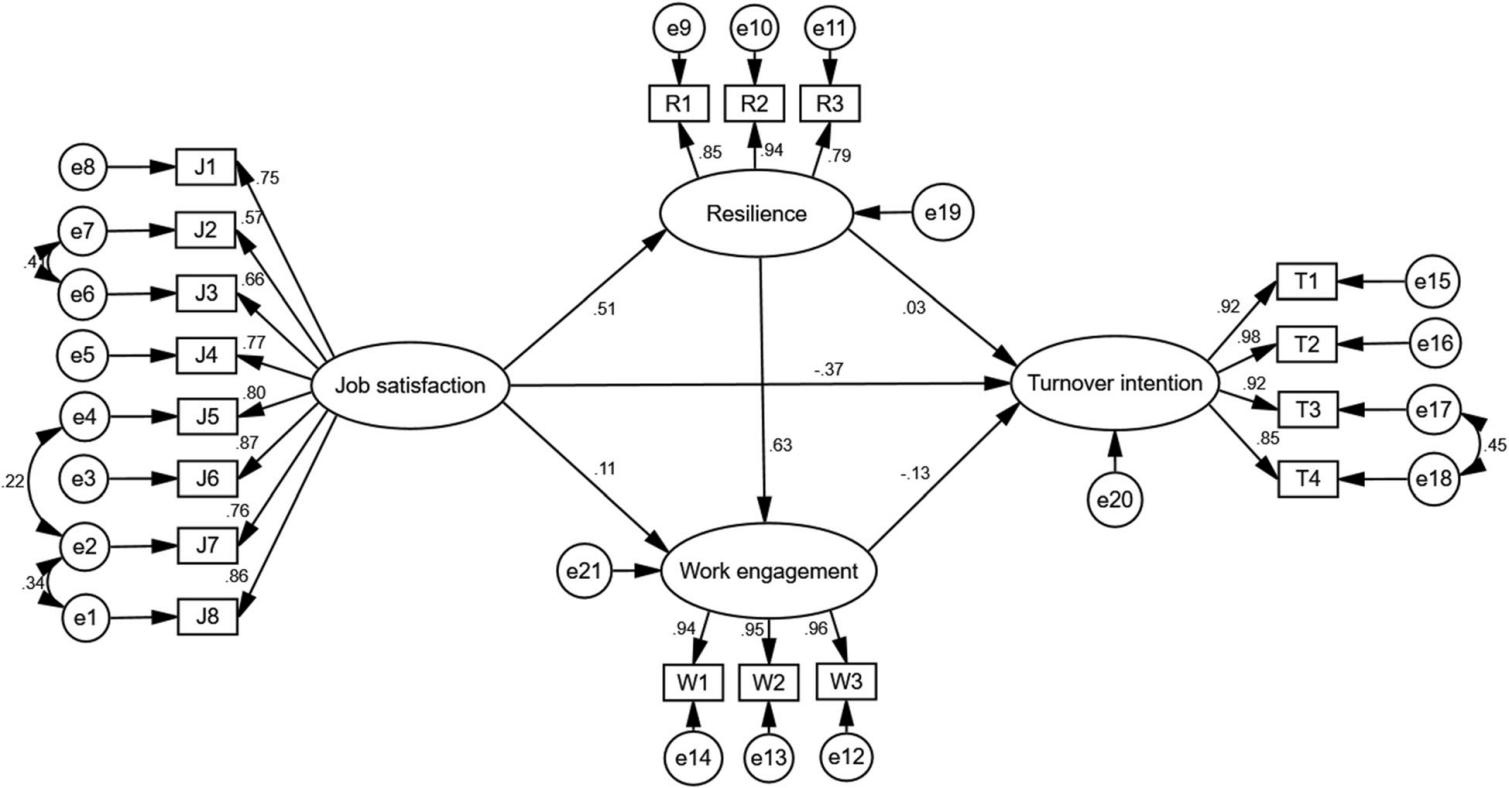
The final model and standardised model paths

The SEM was constructed to interlink and assess the relationship among the four variables (job satisfaction, resilience, work engagement, turnover intention). With generalized least square, the data were fitted to theoretical model, and which was modified according to the model fitting index. The relationship and valid path among four variables were pointed out in the final model (Fig. 2). The final modified hypothetical model’s fit indices were AGFI = 0.911, GFI = 0.935, NFI = 0.964, CFI = 0.966, IFI = 0.966, TLI = 0.959, RMSEA = 0.068, all of which complied with the reference value that presented it as an acceptable model fit.

Each path was guided by 2000 repetitions of Bias-corrected bootstrap using maximum likelihood estimation, and the results of mediation analysis are shown in Table 5. When the 95% confidence interval of the estimated mediating effect does not include 0, it indicates that the mediating effect is statistically significant [7]. Job satisfaction positively affected work engagement (β = 0.11, *p* < 0.001) and resilience (β = 0.51, *p* < 0.001), but a negative effect on turnover intention (β = − 0.37, *p* < 0.001); Work engagement negatively affected turnover intention (β = − 0.13, *p* < 0.001); Resilience had a direct positive effect on work engagement (β = 0.63, *p* < 0.001), but no direct effect on turnover intension (β = 0.03, *p* = 0.138), the final result therefore did not support hypothesis 5 (resilience has a negative effect on turnover intention).

**Table 5:** Significance test of the mediating test

Model Pathways	Estimated	95% CI	Hypothesis
**Total effects**			
Resilience ←Job satisfaction	0.51	0.47–0.55	
Work engagement ←Job satisfaction	0.44	0.39–0.47	
Turnover intention ←Job satisfaction	−0.42	(− 0.46)–(− 0.37)	
Work engagement ←Resilience	0.63	0.59–0.68	
Turnover intention ←Resilience	−0.06	(− 0.11)–(− 0.01)	
Turnover intention ←Work engagement	−0.13	(−0.19)–(− 0.07)	
**Direct effects**			
Resilience ←Job satisfaction	0.51	0.47–0.55	2
Work engagement ←Job satisfaction	0.11	0.07–0.16	3
Turnover intention ←Job satisfaction	−0.37	(−0.42)–(− 0.32)	1
Work engagement ←Resilience	0.63	0.59–0.68	4
Turnover intention ←Resilience	0.03	(−0.04)–0.09	5
Turnover intention ←Work engagement	−0.13	(−0.19)–(− 0.07)	6
**Indirect effects**			
Work engagement ←Job satisfaction	0.32	0.29–0.36	
Turnover intention ←Job satisfaction	−0.04	(−0.07)–(− 0.02)	
Turnover intention ←Resilience	−0.09	(−0.12)–(− 0.04)	

Table 6 shows Significance test of every mediating pathway. Regarding the path between job satisfaction and turnover intention, work engagement had a significant mediate effect, 95%CI:(− 0.03)–(− 0.01), which was consistent with hypothesis 8. But resilience had no mediate effect, 95%CI:(− 0.02)–0.06, which was contrary to hypothesis7. However, in-depth studies had found that resilience played a mediating role between job satisfaction and work engagement, 95%CI:1.90–2.41, (hypothesis 9), and indirectly affected turnover intention through the mediating role of work engagement, 95%CI:(− 0.02)–(− 0.01) (hypothesis 10).

**Table 6:** Significance test of every mediating pathway

Model Pathways	95% CI	Hypothesis
Turnover intention ←Resilience ←Job satisfaction	(− 0.02)–0.06	7
Turnover intention← Work engagement ←Job satisfaction	(−0.03)–(− 0.01)	8
Work engagement← Resilience ←Job satisfaction	1.90–2.41	9
Turnover intention ←Work engagement ←Resilience	(−0.02)–(− 0.01)	10

## Discussion

The purpose of this study is to explore the status of turnover intention of village doctors in China and the effects of job satisfaction, resilience, and work engagement on turnover intention. The unique value of this study lies not only in the selection of village doctors as the research object, but also in the incorporation of these four variables into the structural model for the first time.

The results show that among the 2693 village doctors surveyed, nearly half, 1263 (46.9%) had high turnover intention, which was not only far higher than second-class and higher hospital doctors (6.1%) [47], but also significantly higher than urban community doctors (18.13%) in China [48] and grassroots doctors in other countries, for example, only 11.8% of primary care family physicians had high turnover intention in British [49]. Our study also inquired into the current quality of village doctors. Only 71 (2.6%) had university qualifications or above, and less than 5% had middle and senior professional titles. Although the Chinese government has further lowered the professional title assessment requirements for village doctors, many village doctors still cannot meet the requirements for middle and senior professional titles, which also impacts their income. Chen Zhongqiang, a member of the National Committee of the Chinese People’s Political Consultative Conference (CPPCC), found in a rural survey that with no chance of promotion and low pay, village doctors were not treated as well as veterinarians, and even switched to veterinary medicine [50]. Meanwhile, the aging demographic of doctors has become an international issue, and is even more serious in terms of village doctors due to comparatively less funded rural medical resources. Our survey shows that 1987 (73.7%) of village doctors were 40 years old or above, and only 36 (1.3%) were under 30 years old, hence, the age structure of such doctors is older than that of the doctors in city’s third level hospitals (32.51% aged 40 and over, 17.84% aged 30 and under). At the same time, due to the influence of family structure, health literacy, and lifestyle, the health evaluation age of village doctors is higher than that of urban doctors of the same age, so the retirement of older village doctors is also a reason for high turnover intention. Therefore, more researches are urgently needed to explore the key factors and influencing mechanism of village doctors’ turnover in order to alleviate the phenomenon of village doctors’ massive turnover in China.

The equation model proved whether it was the direct or indirect path effect, village doctors’ job satisfaction contributed the most to turnover intention, which was also mentioned in other studies. A survey of Chinese rural physicians showed that job satisfaction had significant negative effects on turnover intention through work engagement and job burnout as mediators [2]. In our study, village doctors were less satisfied with their jobs, especially in terms of workload, promotion, income, social security, and training opportunities (Table 2). The results conformed with a nationwide survey of village doctors’ mobility which found that 70% of village doctors were unsatisfied with their income, had no hopes for promotion, and lacked old-age security after retirement [47].

The model also demonstrated that work engagement played a mediating role between job satisfaction and turnover intention, and between resilience and turnover intention, which is consistent with other studies. Mi Yu found that, under the direct influence of resilience, work engagement played a mediating role between job environment satisfaction and turnover intention among new nurses [21]. Work engagement is an important evaluation indicator for individual work potential and work efficiency optimization. The improvement of doctors’ work engagement can effectively increase an individual’s physical and mental health, improve job quality, satisfaction and performance, and reduce depression, and thus positively affect the health of patients.

All hypotheses in this study were supported except for hypothesis 5 (The village doctors’ resilience has a negative effect on turnover intention) and hypothesis 7(The village doctors’ job satisfaction has an indirect negative effect on turnover intention through the mediating effect of resilience), and i.e. that resilience had no direct effect on turnover intention, nor did it mediate between job satisfaction and turnover intention, a result contrary to many previous studies [38, 40]. Hodges HF et al. found that enhancing the psychological resilience of nurses in emergency medical center can significantly reduce their turnover rate. Wang M et al. found that the resilience of nurses in first-class tertiary hospitals in China played an intermediary role in job satisfaction and turnover intention. The reason why our finding is different from the above results may be that village doctors mainly deal with frequent and common diseases, and their work stress, work intensity, work trauma and other factors that affect their resilience are lower than hospital nurses, so resilience are not the direct reason for village doctors to leave. However, the study proved that resilience affected turnover intention indirectly through the mediating role of work engagement, and played a significant mediating role in job satisfaction and work engagement. These findings indicated that job satisfaction and work engagement were necessary ways for village doctors to transform the unification of personal value and professional value into continuing their rural medical work. Inspired by these mediation paths, interventions that can sufficiently lower the turnover intention of village doctors should be encouraged. The Chinese government should increase investment in village doctors’ income, establish a long-term, reasonable, and effective promotion mechanism, and provide social security to improve the work efficiency and engagement of village doctors. Another possible strategy is to reduce the work loads of village doctors, improve their resilience, and guide them towards positive coping approaches in their daily work.

In conclusion, our study revealed four affecting paths of turnover intention, and that job satisfaction, resilience, and work engagement were all accurate predictors of the turnover intention of village doctors. Job satisfaction had the most significant, multi-path impact, followed by work engagement. The reason was that resilience cannot directly affect turnover intention, but rather through the mediating effect of work engagement. Hence, this suggests a more sophisticated and in-depth mechanism in the relationship between resilience and turnover intention. However, the research on the resilience of village doctors is very insufficient at present. In the future, we should not only study the influencing factors, but also study the effective intervention measures of the resilience, and construct perfect protective intervention measures of village doctors from the perspective of predictors, so as to improve their resilience. In addition, because resilience is a multi-dimensional, subjective variable, it is difficult to obtain in-depth and detailed understanding through quantitative research. In the future, qualitative research methods should be adopted to conduct micro and in-depth exploration on the resilience of village doctors.

Two limitations of the study should be cleverly addressed. Firstly, although the SEM was used to quantitatively verify the relationship between variables, this study still has limitations to draw definite conclusions based on the cross-sectional design. Secondly, the data were collected through the participants’ self-report questionnaires that were not supervised by researchers and returned through an official letter by Jining Municipal Health Commission, rather than face to face investigation.

## Conclusions

The results show that higher job satisfaction, work engagement, and resilience contribute to reduce turnover intention, which not only provide new ideas to explain the numerous village doctors leaving their jobs, but also offer possible and feasible new methods to reduce turnover intension and behaviour. Future research is proposed to introduce other mediating factors and construct different models to test the influence mechanism of resilience on turnover intention.

In the light of the findings, the health government should actively consider multiple measures to improve the job satisfaction, job engagement, and resilience of village doctors. Work-related dimensions should be paid attention to, including establishing effective promotion mechanisms, increase training opportunities, and providing higher and more reasonable income. Simultaneously, qualitative research should be used to explore the factors influencing resilience in depth and detail, and the protective intervention measures should be structured from the perspective of predictors, so as to improve the resilience of village doctors and their adaptability to stress. In this way, the high turnover rate of village doctors should be effectively contained, and the medical team working in China’s rural primary medical care can develop stably and dynamically.

## Data Availability

The datasets used and/or analyzed during the current study are available from the corresponding author on reasonable request.

## Abbreviations

SD: Standard Deviation
EFA: Exploratory factor analysis
SEM: Structural Equation Modeling
AGFI: Adjust goodness of fit index
NFI: Normed fit index
GFI: Goodness of fit index
CFI: Comparative fit index
TLI: Tucker-Lewis index
IFI: Incremental
RMSEA: Root mean square error of approximation
ANOVA: Analysis of Variance
Cronbach’s α: Cronbach’s Alpha coefficient
